# Recent Advances in Secondary Metabolites from Marine *Aspergillus*

**DOI:** 10.3390/md23100400

**Published:** 2025-10-15

**Authors:** Zimin Wang, Meirong Zhao, Chenglin Li, Yunxia Yu, Zhiqiang Gong, Fandong Kong, Chengzhi Li

**Affiliations:** 1 Key Laboratory of Chemistry and Engineering of Forest Products, School of Chemistry and Chemical Engineering, Education Center for Mental Health, Guangxi Minzu University, Nanning 530006, China; wzm55802023@163.com (Z.W.); yuyunxia2023@163.com (Y.Y.); 2 College of Food and Pharmaceutical Engineering, Guangxi Vocational University of Agriculture, Nanning 530006, China; zhaomeirong124@163.com; 3 Faculty of Chinese Medicine Science, Guangxi University of Chinese Medicine, Nanning 530222, China; mtx569267@163.com

**Keywords:** marine fungi, *Aspergillus*, secondary metabolites, bioactive compounds, drug leads

## Abstract

Marine *Aspergillus* fungi, adapted to extreme marine environments (e.g., sediments, corals, mangroves), are prolific producers of structurally diverse secondary metabolites with significant bioactivities. This review comprehensively analyzes 340 novel natural products reported from 81 marine-derived *Aspergillus* strains over the past three years, classifying them into six major categories: alkaloids (31.2%), polyketides (29.4%), terpenoids, lignans, cyclopeptides, and others. Bioactivity assessments reveal broad therapeutic potential, including antitumor, antimicrobial, anti-inflammatory, and antiviral effects. Notably, marine sediments constitute the primary source (25.9% of strains), followed by sponges and corals. The predominance of alkaloids and polyketides underscores their pharmacological relevance. These findings highlight marine *Aspergillus* as a critical resource for drug discovery, offering promising scaffolds for developing treatments against human diseases and agricultural pathogens.

## 1. Introduction

*Aspergillus* sp., a representative group of marine fungal ecosystems, primarily thrives in typical marine habitats such as mangrove humus substrates, coral reef symbiotic systems, and deep-sea sediments [1]. Its adaptation mechanisms involve systematic responses to extreme physical and chemical conditions, including high osmotic pressure, high hydrostatic pressure, and low dissolved oxygen concentration [2]. Under such selective pressures, marine *Aspergillus* has evolved secondary metabolic pathways distinct from those of its terrestrial relatives through horizontal gene transfer and metabolic network reconstruction, leading to the production of unique natural products [3]. Statistics show that *Aspergillus* fungi are the most studied species among marine microbial-derived new natural products, accounting for 31% of new natural products from marine fungi [4]. These marine natural products exhibit rich chemical diversity, thus facilitating the discovery of drug lead compounds.

The natural products of marine *Aspergillus* show remarkable potential in drug development. Although cases of direct drug approval remain limited, many natural products have advanced the pharmaceutical and agricultural sectors as drug precursors or candidate molecules. The study of the lovastatin metabolic pathway in *Aspergillus terreus* has provided a technical basis for engineering modifications [5]. Currently, candidate drugs have entered the clinical stage: Plinabulin, derived from marine *Aspergillus*, has demonstrated significant efficacy in phase III clinical trials for metastatic non-small cell lung cancer by inhibiting tubulin polymerization and activating immune responses [6]. In agriculture, the novel phenolic aldehyde dimer Stromemycin B from marine *Aspergillus* exhibits potent inhibitory activity against *Ralstonia solanacearum* by inhibiting succinate dehydrogenase activity and disrupting bacterial morphology, significantly reducing disease incidence in a tomato bacterial wilt model [7]. These cases illustrate that although direct drug development based on natural products derived from marine *Aspergillus* still awaits breakthroughs, their core value as lead molecules and semi-synthetic precursors, coupled with the continuous advancement of synthetic biology technologies, is offering innovative solutions for the treatment of major diseases and the development of green agriculture.

**Figure 1:**
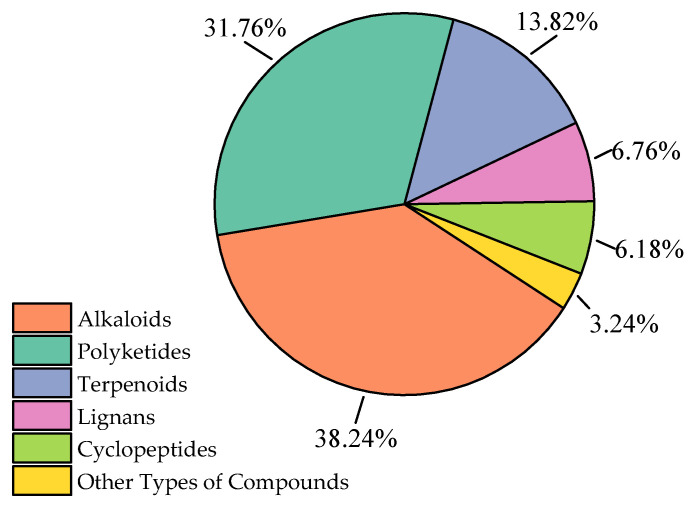
Proportions of compounds with different structures.

Against this backdrop, this study reviews the literature on new natural products derived from marine *Aspergillus* over the past three years, collating 340 new natural products from 81 articles that were first reported to originate from marine *Aspergillus*. In subsequent chapters, these 340 compounds are classified into six categories based on their structural characteristics: alkaloids, polyketides, terpenoids, lignans, cyclopeptides, and other types of compounds. The proportion of each type of compound is shown in Figure 1.

## 2. Compounds

### 2.1. Alkaloids

Alkaloids, nitrogen-containing organic compounds widely distributed in nature, have been increasingly identified from marine-derived *Aspergillus*, in addition to plants, animals, and other microorganisms. Their structures typically feature nitrogen-containing cyclic cores—a characteristic that contributes to remarkable structural diversity, particularly among those isolated from marine *Aspergillus* [8]. Owing to their unique architecures shaped by the extreme marine environment, marine *Aspergillus*-derived alkaloids exhibit prominent and diverse biological activities with significant pharmaceutical and agricultural potential [9,10,11]. Such distinctive properties make marine Aspergillus-derived alkaloids a focal point in natural product chemistry and drug development.

**Figure 2:**
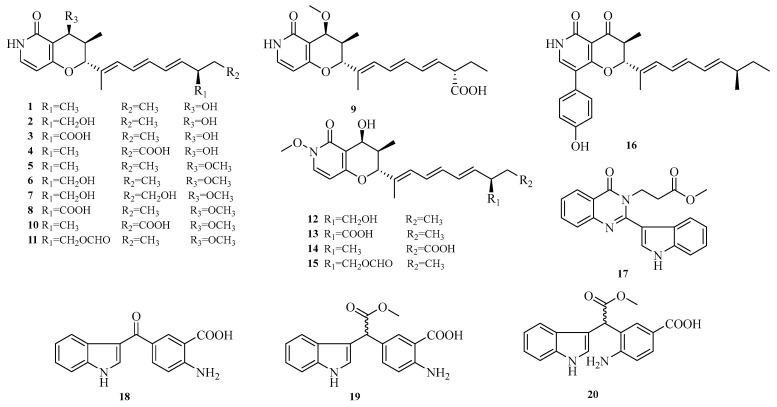
Structural diagrams of compounds **1**–**20**.

Wei-Chen Chen et al. isolated 16 undescribed pyranopyridone alkaloids, aculeapyridones A–P (**1**–**16**), from the co-culture extract of the mangrove-derived fungus *Aspergillus aculeatinus* WHUF0198 and the mangrove-associated *Penicillium* sp. DM27 through bioactivity-guided fractionation. Among them, compounds **12**–**15** with unique N-methoxy groups were identified as activated products of fungal co-culture. The hepatoprotective activity of these compounds against acetaminophen-induced acute liver injury was evaluated in vitro. Results showed that compounds **1**–**7**, **9**, **10**, and **12**–**15** significantly increased cell viability and reduced alanine aminotransferase (ALT) levels in acetaminophen-treated mouse hepatocytes at 5.0 μM or 10.0 μM [12]. Additionally, Lai-Hui Dai et al. reported four new alkaloids (**17**–**20**) isolated from the culture of marine-derived *Aspergillus fumigatus* AF1 [9]. The structures of compounds **1**–**20** are shown in Figure 2. 

Jingshuai Wu et al. investigated the deep-sea sediment-derived fungus *Aspergillus puulaauensis* F77 and successfully isolated **19** undescribed austamide-type diketopiperazines, named versicoines A–S (**21**–**39**). Compound **34** effectively reduced NO production and the expression of iNOS and COX-2 proteins in LPS-induced BV2 cells, suppressed LPS-triggered NF-κB signaling pathway and subsequent NLRP3 inflammasome activation. Compounds **22**, **23**, **36**, and **37** exhibited mild cytotoxicity with cell viability rates of 70.0–75.0% [13].

**Figure 3:**
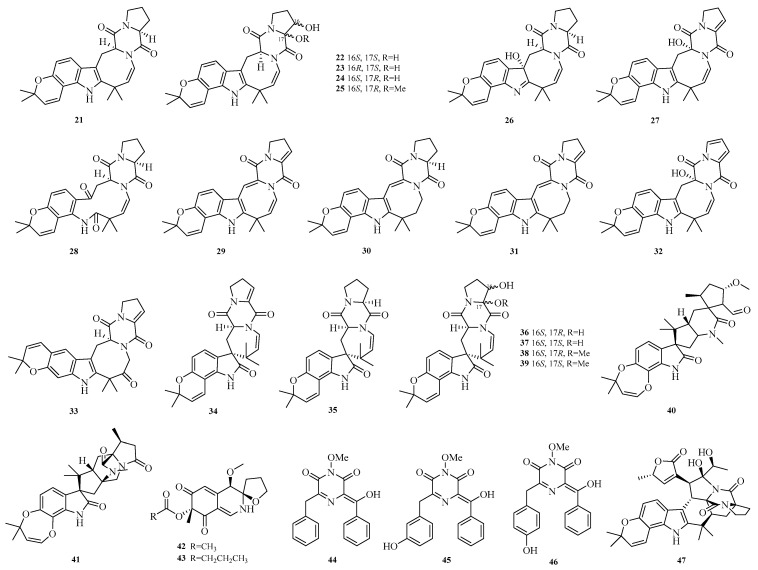
Structural diagrams of compounds **21**–**47**.

Philomina Panin Edjah et al. isolated two new paralectins (PHQ), aculeaquamides B and C (**40**–**41**), from the co-culture of mangrove-derived *Aspergillus aculeatinus* WHUF0198 and mangrove-associated *Penicillium* sp. DM27 [14]. Yao-Yao Zheng et al. studied the sea hare-derived fungus *Aspergillus terreus* RA2905 and identified two alkaloids, azasperones E and F (**42**–**43**) [15]. Sarani Kankanamge et al. cultured the Australian marine sediment-derived fungus *Aspergillus noonimiae* CMB-M0339 and obtained rare 2,6-diketopiperazine alkaloids noonazines A–C (**44**–**46**) [16]. Zheng-Biao Zou et al. isolated a rare stephacidin-asperochratide hybrid, stephaochratidin A (**47**), from deep-sea *Aspergillus ochraceus*. Activity assays showed that stephaochratidin A (**47**) significantly inhibited ferroptosis with an EC_50_ value of 15.4 μM, acting by downregulating heme oxygenase 1 (HMOX-1) expression and suppressing lipid peroxidation [17]. The structures of compounds **21**–**47** are shown in Figure 3. 

Zhibo Hu et al. isolated four diketomorpholine alkaloids (**48**–**51**) and one indole diketopiperazine alkaloid (**52**) from the seagrass-derived *Aspergillus alabamensis* SYSU-6778. Compounds **48** and **49** exhibited potent inhibitory activity against the fish pathogen *Edwardsiella ictalurid*, with minimum inhibitory concentrations (MICs) of 10.0 μM [18]. Yura Ha et al. obtained phthalimidinic acid A (**53**) and phthalimidinic acid B (**54**) from the marine sediment-derived *Aspergillus* sp. ZZ1861. Both compounds showed antifungal activity against *Candida albicans*, with MIC values of 1.6 and 3.1 μM, respectively [19].

**Figure 4:**
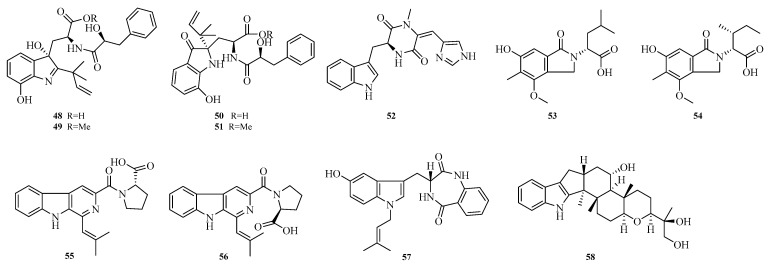
Structural diagrams of compounds **48**–**58**.

Geng-Si Zhang et al. isolated the new natural products secofumitremorgins C (**55**) and D (**56**) from the salt pan-derived *Aspergillus fumigatus* GXIMD00544. Activity assays showed that compound **55** exhibited antifungal spore germination activity against *Fusarium sacchari*-related plant pathogenic fungi, with a 53.0% inhibition rate at 100.0 μM. Additionally, compound **55** demonstrated antifouling potential against *Balanus amphitrite* larval settlement, achieving a 96% inhibition rate at 100.0 μM [20]. Harol Ricardo Arias Cardona et al. reported an undescribed isoprenylated indole derivative, hydroxyhomamide (**57**), from the marine sponge-associated fungus *Aspergillus fischeri* MMERU 23 [21]. Bingying Tang et al. isolated a new ascandinine T (**58**) from the Antarctic sponge-derived fungus *Aspergillus candidus* HDN15-152 [22]. The structures of compounds **48**–**58** are shown in Figure 4. 

Xiaomei Huang et al. isolated three dimeric nitrobenzyl trans-epoxyamides (**59**–**61**) from the culture of deep-sea-derived *Aspergillus terreus* MCCC M28183. Activity assays showed that compound **59** exhibited moderate inhibitory activity against human gastric cancer cell line MKN28 with an IC_50_ value below 10.0 μM [23]. Hao-Yu Yu et al. reported three new oxyindole diterpenoid alkaloids, emeniveol B–D (**62**–**64**), from the marine sediment-derived *Aspergillus* sp. MCCC 3A00392 [24]. Elisa Doro-Goldsmith et al. isolated a novel tryptophan derivative, 12*S*-deoxynorquinoline (**65**), from the marine ascidian-derived fungus *Aspergillus clavatus* AS-107 [25].

Dina H El-Kashef et al. isolated a new isoprenylated indole diketopiperazine alkaloid, rubrumline P (**66**), from the fermented culture of *Aspergillus chevalieri*, a marine sediment-derived fungus collected at a depth of 15 m near the Lighthouse of Dahab, Red Sea, Egypt. Compound rubrumline P (**66**) was confirmed to exhibit cytotoxic activity against PANC-1 cancer cells with an IC_50_ value of 25.8 μM. Although the underlying mechanism remains elusive, cell cycle analysis showed a slight increase in the sub-G1 peak following treatment with compound **66** [26]. Cangzhu Sun et al. emphasized the importance of fungi as a source of novel bioactive natural products and isolated asperindopiperazines A–C (**67**–**69**) from Mariana Trench-associated *Aspergillus* sp. SY2601 [27]. Yi-Hao Che et al. isolated a novel diketopiperazine derivative, 8*R*-methoxy-9*R*-hydroxy-fumigaclavine C (**70**), from *Aspergillus fumigatus* CYH-5 collected from a seahorse cold seep [28].

**Figure 5:**
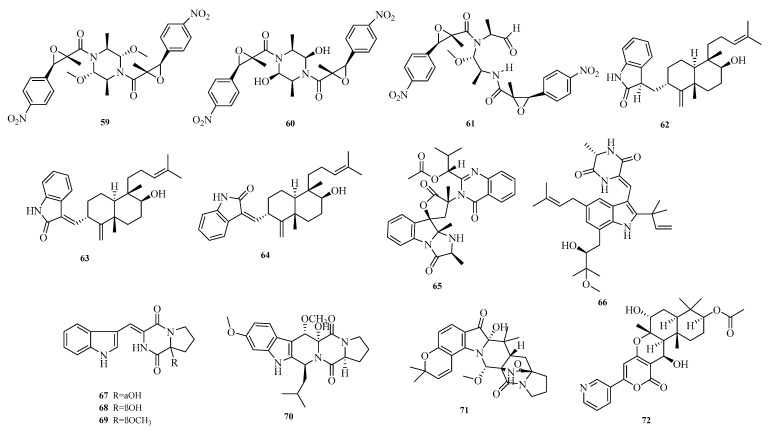
Structural diagrams of compounds **59**–**72**.

Yu Chen et al. isolated and identified a new derivative, aspertaichamide A (**71**), from the endophytic fungus *Aspergillus taichungensis* 299 derived from the marine red alga *Gelidium amansii*. In vitro cytotoxicity assays showed that new compound **71** reduced AGS cell viability in a concentration-dependent manner, with an IC_50_ value of 1.7 μM. Further studies indicated that **71** might induce programmed cell death in AGS cells via an apoptotic pathway [29]. Zhu Chen et al. obtained a new alkaloid, pyripyropene U (**72**), from the marine sponge-derived *Aspergillus* sp. SCSIO41420 [30]. The structures of compounds **59**–**72** are shown in Figure 5. 

Mangaladoss Fredimoses et al. isolated two new alkaloids, chaetominines A (**73**) and B (**74**), from the marine sponge-derived fungus *Aspergillus versicolor* SCSIO XWS04 F52. Activity assays showed that compounds **73** and **74** exhibited cytotoxic activity against leukemia K562 and colon cancer SW1116 cells, with half-maximal inhibitory concentration (IC_50_) values ranging from 7.5 to 12.5 μM [31]. Shui-Hua Lin et al. conducted a systematic chemical study on the deep-sea-derived fungus *Aspergillus versicolor* 170217, isolating three new alkaloids: citriquinolinone A–B (**75**–**76**) and *N*-(3-((2′*S*,3′*S*)-2′,3′,5′-trimethyl-1,3-dioxolan-2-yl)propyl)acetamide (**77**) [32].

**Figure 6:**
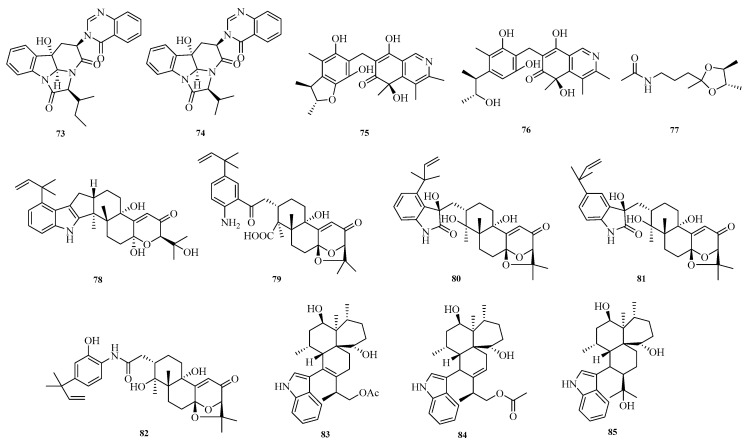
Structural diagrams of compounds **73**–**85**.

Fei Zhang et al. isolated and identified the fungus *Aspergillus* sp. ZF-104 from marine soft corals collected in Haikou Bay, China. Eight undescribed indole-diterpenoid alkaloids, penerpenes O–V (**78**–**85**), were isolated and characterized from this strain. The inhibitory activity of these compounds against protein tyrosine phosphatase 1B (PTP1B) was evaluated. In the PTP1B inhibition assay, compounds **78**, **79**, and **84** showed activities comparable to that of the positive control [33]. The structures of compounds **73**–**85** are shown in Figure 6. 

**Figure 7:**
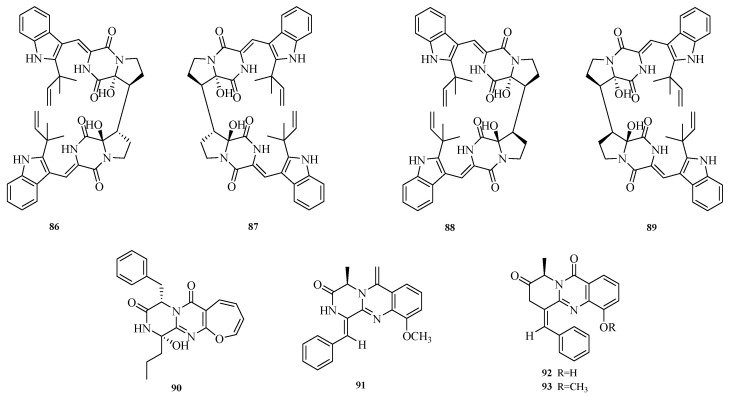
Structural diagrams of compounds **86**–**93**.

Ying-Jie Zhao et al. obtained two pairs of new dimeric diketopiperazine alkaloids, (±)-dibrevianamides Q1 and Q2 ((±)-1 and (±)-2) (**86**–**89**), from marine-derived *Aspergillus* sp. Activity assays showed that compounds **86** and **89** exhibited resistance to H1N1 virus with half-maximal inhibitory concentration (IC_50_) values of 12.6 and 19.5 μM, respectively. Compound **86** also demonstrated significant activity against *Mycobacterium tuberculosis* with a minimum inhibitory concentration (MIC) of 10.2 μM [34]. Qi Hong et al. conducted a secondary metabolite study on the filamentous fungus *Aspergillus puniceus* FAHY0085 isolated from a South China Sea coral sample, isolating four undescribed alkaloids, including an oxyepin-containing diketopiperazine-type alkaloid (**90**) and three 4-quinazolone alkaloids (**91**–**93**). The transcriptional activation of liver X receptor α (LXRα) by the isolated compounds was evaluated. Results showed that the new diketopiperazine puniceloid F (**92**) exhibited significant transcriptional activation activity against LXRα with half-maximal effective concentration (EC_50_) values ranging from 2.0 to 15.0 μM [35]. The structures of compounds **86**–**93** are shown in Figure 7. 

Komal Anjum et al. isolated a novel alkaloid with a pyridoindole hydroxymethylpiperazine dione structure, aspergill alkaloid A (**94**), from the deep-sea-derived fungus *Aspergillus* sp. HDN20-1401. Antibacterial assays showed that aspergill alkaloid A (**94**) exhibited inhibitory activity against Bacillus cereus with a minimum inhibitory concentration (MIC) of 12.5 μM [36]. Yu-Liang Dong et al. isolated multiple compounds from *Aspergillus versicolor* AS-212, an endophyte derived from the deep-sea coral *Hemicorallium* cf. *imperiale* collected from the Magellan Seamounts in the Western Pacific. The isolation yielded four new oxazine-based pyrimidine alkaloids, versicoxepines A–D (**95**–**98**), and two quinolinone analogs: 3-hydroxy-6-methoxy-4-phenylquinolin-2(1*H*)-one (**99**) and 3-methoxy-6-hydroxy-4-phenylquinolin-2(1*H*)-one (**100**). Activity tests showed that compound **99** exhibited antibacterial activity against aquatic pathogens *Vibrio harveyi* and *V. alginolyticus* with an MIC of 8.0 μM [37].

Yu-Liang Dong et al. isolated and identified two new quinazoline diketopiperazine alkaloids, versicomide E (**101**) and cottoquinazoline H (**102**), from the endophytic fungus *Aspergillus versicolor* AS-212 associated with deep-sea corals. In antibacterial assays, compound **102** exhibited inhibitory effects against *Vibrio harveyi* and *Vibrio parahaemolyticus* with an MIC of 9.0 μM [38].

**Figure 8:**
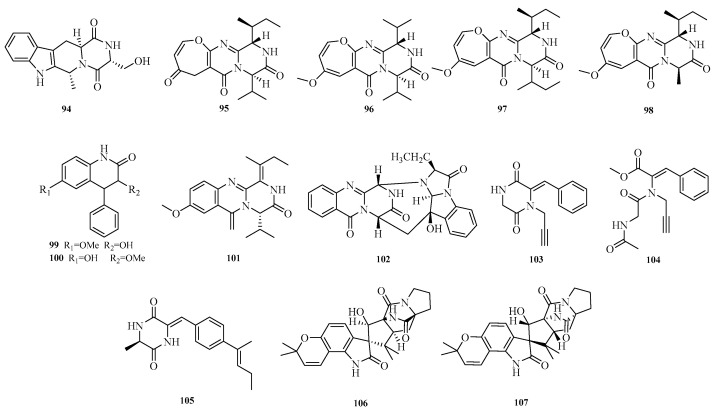
Structural diagrams of compounds **94**–**107**.

Jun-Qiu Mao et al. studied the marine-derived fungus *Aspergillus sclerotiorum* ST0501 from the South China Sea, from which three new alkaloids, sclerotioloids A–C (**103**–**105**), were obtained. Sclerotioloid B (**104**) showed inhibition of LPS-induced NO production, with an inhibition rate 28.9% higher than that of dexamethasone (25.9%) [39]. Jin-Shan Hu et al. isolated two new indolediketopiperazine alkaloids (IDAs), namely (+)-19-epi-sclerotiamide (**106**) and (−)-19-epi-sclerotiamide (**107**), from the epiphytic fungus *Aspergillus versicolor* CGF9-1-2 associated with soft corals [40]. The structures of compounds **94**–**107** are shown in Figure 8. 

Li-Hong Yan et al. isolated and characterized five new antibacterial indolediketopiperazine alkaloids from a deep-sea cold seep-derived *Aspergillus chevalieri*, namely 24,25-dihydroxyvariecolorin G (**108**), 25-hydroxyrubrumazine B (**109**), 22-chloro-25-hydroxyrubrumazine B (**110**), 25-hydroxyvariecolorin F (**111**), and 27-epi-aspechinulin D (**112**). Activity assays showed that compounds **108**–**112** exhibited inhibitory activity against various pathogens with minimum inhibitory concentration (MIC) values ranging from 4.0 to 32.0 μM [41]. Zhibo Hu et al. isolated and identified six new benzoic acid-containing alkaloids from seagrass-derived *Aspergillus candidus*, namely asperalins A–D (**113**–**116**), asperaluhalazine A (**117**), and *N*-(3-acetamidopropyl)-3,4-dihydroxybenzamide (**118**). Compounds **115** and **116** showed strong activity against *Staphylococcus aureus*, *Streptococcus* iniae, and *Streptococcus parauberis*, with MIC values of 10.1, 5.0 and 10.1 μM, respectively [42].

Florent Magot et al. isolated 77 microbial strains from the seafloor at a depth of 2454 m in the Fram Strait, Arctic Ocean. Using the one-strain-many-compounds (OSMAC) cultivation method, they isolated a new polyketide synthase-nonribosomal peptide synthetase (PKS-NRPS) hybrid macrolide, heteroamidin A (**119**), and a new quinazoline, (−)-isocarbonolide A (**120**) [43]. Xinyang Li et al. isolated two new dimeric diketopiperazine stereoisomers (**121**–**122**) from the culture broth of an *Aspergillus* strain derived from the intestine of Lip Tarico [44]. Zhong-Hui Huang et al. isolated one previously undescribed compound, punicesterones A (**123**), from the deep-sea-derived fungal strain *Aspergillus puniceus* SCSIO z021 [45].

**Figure 9:**
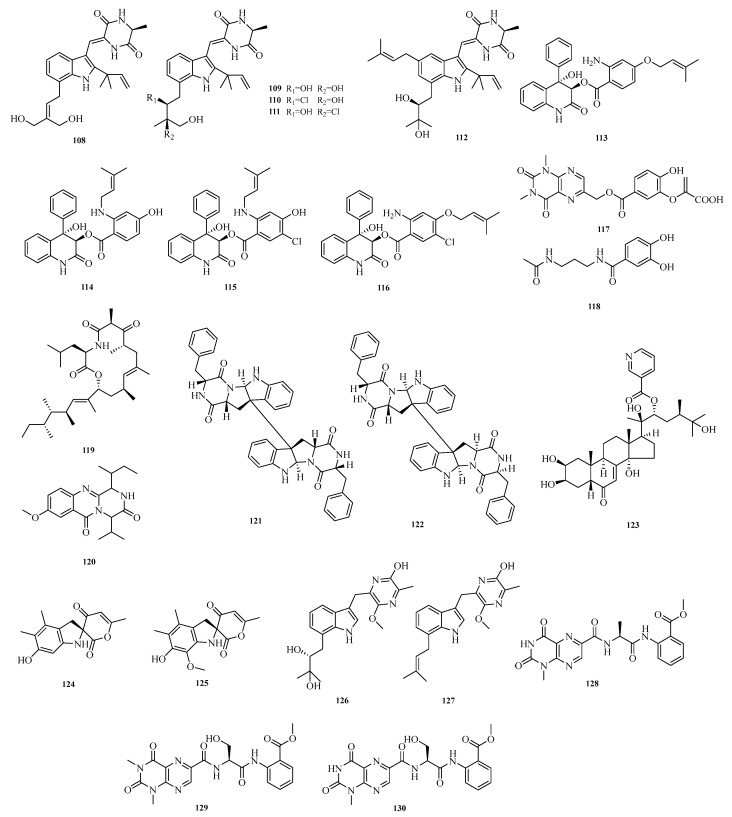
Structural diagrams of compounds **108**–**130**.

Yao-Yao Zheng et al. conducted a chemical study on the marine sediment-derived fungus *Aspergillus terreus* PPS1, successfully isolating and identifying seven previously undescribed alkaloids, namely asperspiroids A and B (**124**–**125**), astepyrazinol C (**126**); two luhalazine peptides, scytalols C and D (**127**–**128**); and scytalols E and F (**129**–**130**). Activity evaluation results showed that astepyrazinol C (**126**) exhibited significant inhibitory activity against lipopolysaccharide (LPS)-induced nitric oxide (NO) production in RAW264.7 macrophages, with an inhibition rate of 37.4% at 20.0 μM [46]. The structures of compounds **108**–**130** are shown in Figure 9. 

The sources and biological activities of compounds **1**–**130** are summarized in Table 1.

### 2.2. Polyketides

Polyketide compounds are a family of natural products synthesized by polyketide synthases (PKSs). Their carbon skeletons are formed through modular extension of acetyl/malonyl units [47]. Depending on the type of PKS (Type I, II, or III), these compounds can form linear, cyclic, or highly modified complex structures, including macrolides (e.g., erythromycin), aromatic polyketides (e.g., tetracycline), and polyethers (e.g., amphotericin B). Their structural diversity arises from combinations of post-modification reactions such as ketone reduction, cyclization, and methylation. These compounds exhibit broad-spectrum biological activities.

**Figure 10:**
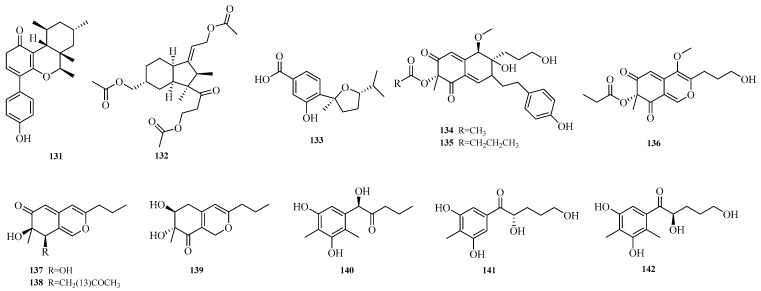
Structural diagrams of compounds **131**–**142**.

Wei-Chen Chen et al. isolated one undescribed polyketide, aculeapyridones Q (**131**), from the co-culture extract of mangrove-derived fungus *Aspergillus aculeatinus* WHUF0198 and mangrove-associated fungal *Penicillium* sp. DM27 via bioactivity-guided fractionation [12]. Yue Jiang et al. isolated asperhydrindane A (**132**) from the mangrove-derived fungus *Aspergillus terreus* GXIMD 03,158 [48]. Xu-Meng Ren et al. isolated (7*R*,10*R*)-11-dehydroxy-iso-10-hydroxysydowic acid (**133**) from the deep-sea-derived fungus *Aspergillus sydowii* DFFSCS007 [49]. Yao-Yao Zheng et al. conducted a study on the sea hare-derived fungus *Aspergillus terreus* RA2905, identifying nine new polyketide compounds, namely azasperones C–D, G–J (**134**–**139**) and preazasperones A–C (**140**–**142**) [15]. The structures of compounds **131**–**142** are shown in Figure 10. 

Yu-Pei He et al. conducted a study on the marine fungus *Aspergillus versicolor* CGF9-1-2, aiming to discover undescribed compounds. They successfully isolated four new polyketides, including decumbenone E (**143**), decumbenone F (**144**), 2′-epi-8-*O*-methylnidurufin (**145**), and (−)-phomoindene A (**146**). In vitro screening for TDP1 inhibitory activity of all isolated compounds showed that compound **145** exhibited weak inhibitory activity against TDP1 with an IC_50_ value of 33.00±5.10 μM [50]. Ailiman Abulaizi et al. isolated a marine-derived fungal strain *Aspergillus* sp. ITBBc1 from corals collected in the South China Sea, Hainan Province. In-depth chemical investigation of the fermented extract of this strain yielded four new secondary metabolites (**147**–**150**), named megastigmanones A-C and prenylterphenyllin H [51]. Sarani Kankanamge et al. performed culture analysis on *Aspergillus noonimiae* CMB-M0339, a fungus derived from Australian marine sediments, obtaining a new aza-nonaketide, noonaphilone A (**151**) [16].

**Figure 11:**
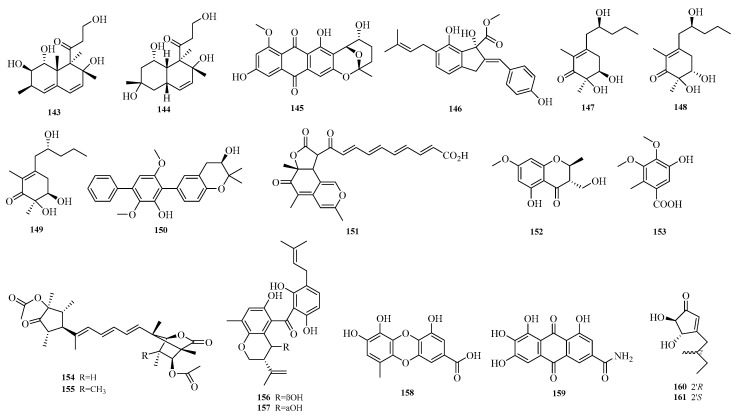
Structural diagrams of compounds **143**–**161**.

Zhibo Hu et al. isolated one chromone (**152**) and one benzoic acid derivative (**153**) from the seagrass-derived *Aspergillus alabamensis* SYSU-6778 [18]. Yura Ha et al. obtained emericelactones F and G (**154**–**155**), 20*R*,25*S*-preshamixanthone (**156**), 20*R*,25*R*-preshamixanthone (**157**), aspergilol G (**158**), and 2-hydroxyemodic amide (**159**) from the marine sediment-derived *Aspergillus* sp. ZZ1861. Aspergilol G (**158**) and 2-hydroxyemodic amide (**159**) exhibited antifungal activity against Candida albicans with minimum inhibitory concentration (MIC) values of 1.6 and 3.1 μM, respectively [19]. Guang-Yu Zhang et al. identified terreins A and B (**160**–**161**) from the coral-derived fungus *Aspergillus terreus* [52]. The structures of compounds **143**–**161** are shown in Figure 11. 

Yi-Hao Che et al. isolated seven new phenol derivatives, namely subversins A–E (**162**–**166**), subversic acid A (**167**), and epi-wortmannine G (**168**), as well as one new natural product 4-hydroxy-7-methoxyphthalide (**169**), from the fungus *Aspergillus subversicolor* CYH-17 collected from a seahorse cold seep [53]. Chun-Ju Lu et al. isolated six benzophenone derivatives, carneusones A–F (**170**–**175**), from the marine sponge-derived fungal strain *Aspergillus carneus* GXIMD00543. Using lipopolysaccharide (LPS)-induced RAW 264.7 cells, they evaluated the effect of these compounds on nitric oxide (NO) secretion. The results showed that compounds **174** and **175** exhibited moderate anti-inflammatory activity with half-maximal effective concentration (EC_50_) values of 34.6 ± 0.9 and 20.2 ± 1.8 μM, respectively [54]. Hao-Yu Yu et al. isolated two new polyketides, hamavellone C and (+)-Stagonospone A (**176**-**177**), from the marine sediment-derived *Aspergillus* sp. MCCC 3A00392. Highlighting the importance of fungi as a source of novel bioactive natural products [24]. Cangzhu Sun et al. isolated 5-methoxy-8,9-dihydroxy-8,9-deoxyaspyrone (**178**) from the Mariana Trench-related *Aspergillus* sp. SY2601 [27].

**Figure 12:**
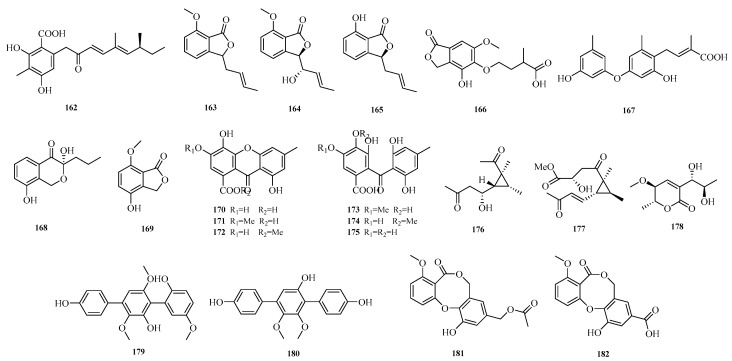
Structural diagrams of compounds **162**–**182**.

Yanbo Zeng et al. obtained two undescribed compounds, asperterphenylcins A–B (**179**–**180**), and another two undescribed compounds, asperdiphenylcins A–B (**181**–**182**), from the marine-derived fungus *Aspergillus candidus* HM5-4 isolated from a South China Sea sponge. Activity assays showed that compound **179** exhibited strong inhibitory activity against *Neoscytalidium dimidiatum*, with an inhibition zone diameter of 31.7 ± 2.6 mm at a concentration of 10.0 μg/disk; compound **180** displayed potent inhibitory activity against *α*-glucosidase, with an IC_50_ value of 1.3 ± 0.2 μM [55]. The structures of compounds **162**–**182** are shown in Figure 12. 

Jingjing Xue et al. successfully isolated three phenolic compounds, namely carnemycin H–I (**183**–**184**) and stromemycin B (**185**), from secondary metabolites of a marine-derived *Aspergillus strain*. Antibacterial activity evaluation of the isolated compounds against *Ralstonia solanacearum* (bacterial wilt pathogen) showed that compound **185** exhibited excellent inhibitory activity with a minimum inhibitory concentration (MIC) of 3.0 μM, which was comparable to that of streptomycin sulfate. Furthermore, compound **185** significantly altered the morphology of *R. solanacearum* and inhibited the activity of succinate dehydrogenase (SDH), thereby interfering with the growth of R. solanacearum [7].

Chao Li et al. conducted a study on the starfish-derived fungus *Aspergillus* sp. WXF1904, isolating one new brominated isocoumarin, namely 5-bromo-6,8-dihydroxy-3,7-dimethylisocoumarin (**186**), as well as four new natural products: methyl 3-bromo-2,4-dihydroxy-6-methylbenzoate (**187**), methyl 2-bromo-4,6-dihydroxybenzoate (**188**), (*E*)-3-(3-bromo-4-hydroxyphenyl)acrylic acid (**189**), and 4-hydroxy-3-methyl-6-phenyl-2*H*-pyran-2-one (**190**). Evaluation of the acetylcholinesterase and pancreatic lipase inhibitory activities of these compounds showed that the new compound **186** exhibited weak inhibitory activity against acetylcholinesterase, while compounds **187** and **190** displayed weak inhibitory activity against pancreatic lipase [56].

Ying Chen et al. conducted a study on the coral-derived fungus *Aspergillus austwickii* SCSIO41227 from the Beibu Gulf, obtaining three previously uncharacterized compounds, asperpentenones C–E (**191**–**193**). Bioassay results showed that compound **191** exhibited significant NA inhibitory activity with a half-maximal inhibitory concentration (IC_50_) of 31.3 μM, while compound **192** displayed weak inhibitory activity against PL [57]. Mangaladoss Fredimoses et al. isolated a new oxytetracycline derivative (**194**) from the marine sponge-derived fungus *Aspergillus versicolor* SCSIO XWS04 F52 [31]. Shui-Hua Lin et al. performed a systematic chemical investigation on the deep-sea-derived fungus *Aspergillus versicolor* 170217, isolating (6,8-dihydroxy-4-methyl-1-oxo-1*H*-isochromen-3-yl)methyl (**195**) [32].

**Figure 13:**
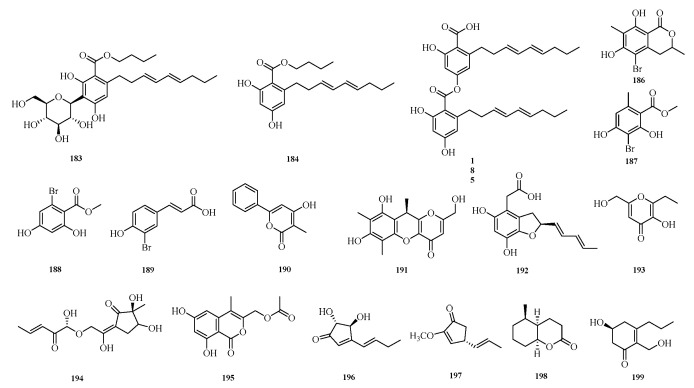
Structural diagrams of compounds **183**–**199**.

Youmin Ying et al. isolated the fungus *Aspergillus terreus* F6-3 from the body surface of Johnius belangerii collected from the coastal waters of Hainan Province, China. From this fungal strain, they identified two previously undescribed compounds, asperterreinones A–B (**196**–**197**), and one new compound, (±)-asperterreinin A (**198**) [58]. Zhibo Hu et al. isolated a new cyclohexanone derivative, insuetone A (**199**), from the seagrass-derived fungus *Aspergillus insuetus* SYSU6925. Activity assays showed that compound **199** exhibited weak to moderate antifungal activity against four phytopathogenic fungi, with minimum inhibitory concentration (MIC) values in the range of 50.0 μM [59]. The structures of compounds **183**–**199** are shown in Figure 13. 

Weibo Zhao et al. isolated three new phenolic compounds, namely epicocconigrones C–D (**200**–**201**) and flavimycin C (**202**), from the fermentation culture of a deep-sea sediment-derived fungus *Aspergillus insulicola*. Evaluation of their *α*-glucosidase inhibitory activity showed that compound **200** exhibited strong inhibitory effect on *α*-glucosidase with a half-maximal inhibitory concentration (IC_50_) of 17.0 μM, which was significantly higher than that of the positive control acarbose (IC_50_ = 823.0 μM). This indicates that compound **200** has the potential to be a promising lead compound for new hypoglycemic drugs [60]. Jun Wu et al. isolated five new dimeric tetrahydroanthraquinones, aculeaxanthones A–E (**203**–**207**), from the fungus *Aspergillus aculeatinus* WHUF0198. Compound **205** showed cytotoxicity against the Bel-7402 cell line (IC_50_ = 2.0 μM) [61].

**Figure 14:**
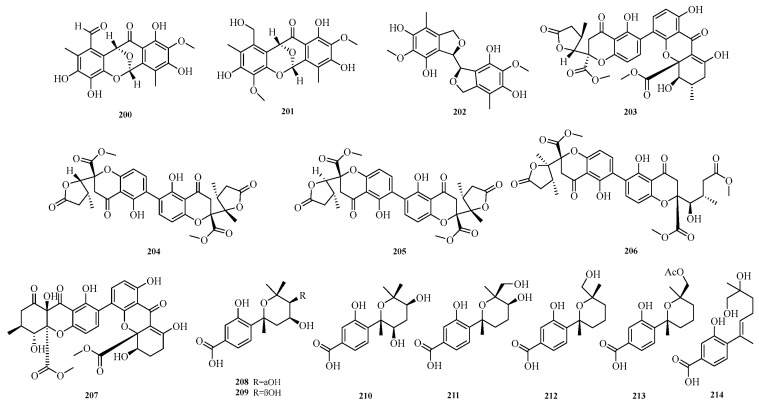
Structural diagrams of compounds **200**–**214**.

Yuanli Li et al. successfully obtained seven new phenolic bisabolane sesquiterpenoids (**208**–**214**) from the deep-sea-derived fungus *Aspergillus versicolor* YPH93. Evaluation of the effects of all compounds on ferroptosis showed that compound **214** exerted an inhibitory effect on erastin/RSL3-induced ferroptosis, with a half-maximal effective concentration (EC_50_) in the range of 2.0 to 4.0 μM [62]. The structures of compounds **200**–**214** are shown in Figure 14. 

Xin Qi et al. conducted a study on the sponge-derived fungus *Aspergillus* sp. SCSIO41315, from which they isolated 21 new terphenyl derivatives, namely asperterphenyls A-N and the enantiomers of asperterphenyls B–H (**215**–**235**). Activity assays revealed that asperterphenyl A (**215**) exhibited neuraminidase inhibitory activity with a half-maximal inhibitory concentration (IC_50_) of 1.8 ± 0.5 μM, and it could effectively inhibit infections by various H1N1 virus strains with IC_50_ values ranging from 0.7 ± 0.3 to 1.5 ± 0.6 μM. Its mechanism of action involves reducing virus plaque formation in a dose-dependent manner, indicating that asperterphenyl A (**215**) holds potential as a promising antiviral compound in the pharmaceutical field [63].

**Figure 15:**
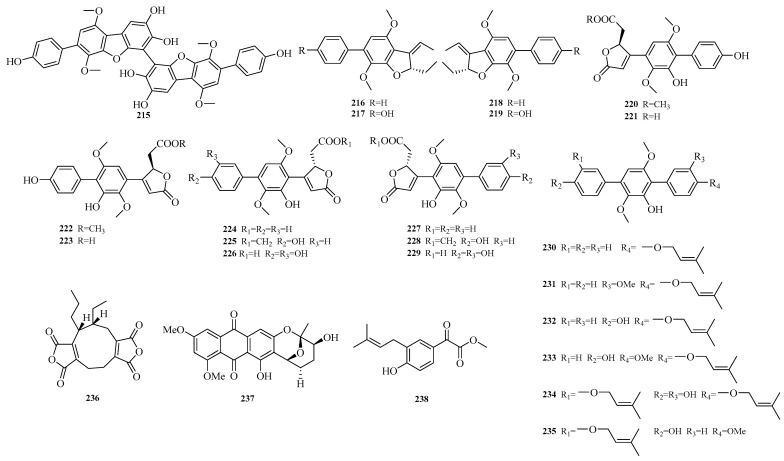
Structural diagrams of compounds **215**–**238**.

Baiq Nila Sari Ningsih et al. isolated a new nonapeptide enantiomer, ent-epiheveadride (**236**), from the marine-derived fungus *Aspergillus chevalieri* PSU-AMF79. Activity assays showed that compound **236** exhibited antifungal activity against *Cryptococcus neoformans* ATCC90113 (flucytosine-resistant) and *Candida albicans* NCPF3153, with minimum inhibitory concentration (MIC) values of 128.0 μM and 200.0 μM, respectively [64]. Ze’en Xiao et al. isolated a new anthraquinone, asperquinone A (**237**), from the mangrove endophytic fungus *Aspergillus* sp. 16-5C [65]. Xin Qi et al. isolated a new glyoxylate-containing benzene derivative, 2-(4-hydroxy-3-(3′-methyl-2′-butenyl)phenyl)-2-oxoacetate (**238**), from the marine alga-derived fungus *Aspergillus* sp. SCSIO 41,304 [66]. The structures of compounds **215**–**238** are shown in Figure 15. 

The sources and biological activities of compounds **131**–**238** are summarized in Table 2.

### 2.3. Terpenoids

Terpenoids are naturally occurring organic compounds widely distributed in nature. Composed of covalently linked isoprene units, they form unique molecular skeletons with remarkable structural diversity; they are further classified by the number of isoprene units into categories such as monoterpenes, sesquiterpenes, and diterpenes. These compounds are primarily derived from plants, though some are also biosynthesized by microorganisms and marine organisms. Endowed with unique properties and broad application prospects, terpenoids have become a research hotspot across multiple fields and are expected to drive the advancement of future pharmaceuticals [67].

Xu-Meng Ren et al. isolated two new terpenoid derivatives, (1*S*,6*R*,7*S*)-hydrobenzosydowic acid (**239**) and (1*R*,6*S*,7*S*)-hydrobenzosydowic acid (**240**), from the deep-sea-derived fungus *Aspergillus sydowii* DFFSCS007 [49]. Guang-Ping Cao et al. identified a new compound, millmerranones G (**241**), from the mangrove-derived fungus *Aspergillus* sp. GXIMD 03004. This fungus was isolated from the leaves of the mangrove plant Acanthus ilicifolius L. collected from the Beibu Gulf, China. Evaluation of anti-Vibrio activity showed that compound **241** exhibited weak activity against Vibrio harveyi [68].

Zhen Zhang et al. isolated 10 new ergot derivatives (**242**–**251**) from the deep-sea-derived fungus *Aspergillus terreus* YPGA10. Compound **242** exhibited cytotoxicity against the human colon cancer SW620 cell line with an IC_50_ value of 8.4 μM. It also showed cytotoxicity against five human leukemia cell lines (CCRF-CEM, Jurkat, THP-1, U937, and K562) with IC_50_ values ranging from 5.0 to 9.0 μM. Compound **250** displayed weak inhibitory activity against RSL3-induced ferroptosis in U937 cells, with an EC_50_ value of 30.0 μM [69]. Jun Zhang et al. discovered two new compounds with a very rare structural skeleton, asperporonins A (**252**) and B (**253**), from the deep-sea fungus *Aspergillus terreus* SCSIO 41,202 [70].

**Figure 16:**
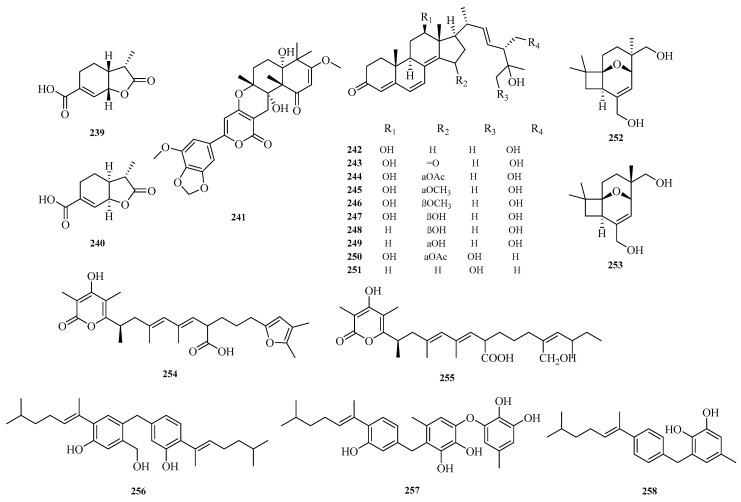
Structural diagrams of compounds **239**–**258**.

Yiwei Hu et al. isolated two new 6-alkenyl pyrone polyketides, alternapyrones G-H (**254**–**255**), from the marine-derived fungal strain *Arthrinium arundinis*. Activity assays revealed that alternapyrone G (**254**) not only inhibited M1 polarization in lipopolysaccharide (LPS)-stimulated BV2 microglia but also promoted dendritic regeneration and neuronal survival after Aβ treatment. This indicates that compound **254** has the potential to serve as a privileged scaffold for the development of anti-Alzheimer’s disease drugs [71]. Hao-Yu Yu et al. isolated a rare dimeric aromatic bisabolane sesquiterpenoid, aspergol A (**256**), and two undescribed phenolic bisabolane sesquiterpenoids, expansol H and aspergol B (**257**–**258**), from the marine sediment-derived *Aspergillus* sp. MCCC 3A00392 [24]. The structures of compounds **239**–2**58** are shown in Figure 16. 

Cangzhu Sun et al. focusing on the significance of fungi as a source of novel bioactive natural products, isolated 12*S*-aspertetranone D (**259**) from the Mariana Trench-related *Aspergillus* sp. SY2601. The new compound **259** exhibited antibacterial activity against both methicillin-resistant *Staphylococcus aureus* and *Escherichia coli*, with MIC values of 3.8 μM and 5.0 μM, respectively [27]. Hui Cui et al. isolated six previously undescribed salinene-type terpenoids, aspertermeroterpenes A–F (**260**–**265**), from the marine-derived fungus *Aspergillus terreus* GZU-31-1. During the bioactivity assay, it was found that aspertermeroterpene B (**261**) effectively inhibited the activation of hepatic stellate cells at a concentration of 5.0 μM by targeting the Nrf2 signaling pathway. This is the first report that aspertermeroterpene B, as a newly discovered carbon skeleton of meroterpenoids, possesses anti-hepatic fibrosis activity [72].

Ying Chen et al. conducted a study on the coral-derived fungus *Aspergillus austwickii* SCSIO41227 from the Beibu Gulf, obtaining asperpentenone B (**266**) [57]. Shui-Hua Lin et al. performed a systematic chemical investigation on the deep-sea-derived fungus *Aspergillus versicolor* 170217, isolating two new dimeric citrinin analogs, dicitrinones K–L (**267**–**268**) [32]. Hui-Min Wen et al. isolated a new compound, 3*β*-hydroxy-5*α*,6*β*-methoxyergosta-7,22-dien-15-one (**269**), from the crude extract of a marine sponge-derived *Aspergillus* sp. Antibacterial activity evaluation showed that compound **269** exhibited antibacterial activity against Staphylococcus aureus [73].

**Figure 17:**
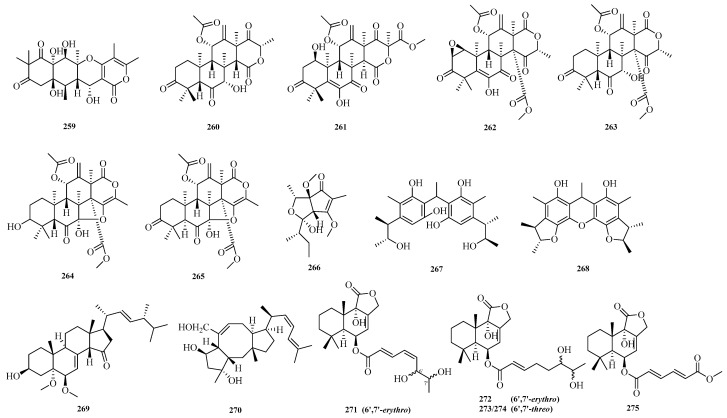
Structural diagrams of compounds **259**–**275**.

Sheng-Tao Fang et al. conducted a chemical investigation on the marine alga-derived strain *Aspergillus* sp. RR-YLW-12, identifying six new terpenoids: 21-Deoxo-21-hydroxyophiobolin U (**270**) and ustusolates K–O (**271**–**275**). The growth inhibitory effects of all compounds against five species of harmful marine microalgae were evaluated. The new compounds exhibited significant to moderate inhibitory effects on all tested microalgal species, with IC_50_ values ranging from 5.8 to 54.5 μM [74]. The structures of compounds **259**–**275** are shown in Figure 17. 

Xinjun Zhang et al. identified a new bisabolane-type sesquiterpenoid, named (+)-8-dehydroxylaustrosene (**276**), from the fungus *Aspergillus sydowii* (BTBU20213012) isolated from marine sediment samples in the Western Pacific Ocean [75]. Mohamed S. Elnaggar et al. isolated a new thioterpenoid, austalide Z (**277**), from a soft coral-associated fungus. In vitro cytotoxicity evaluation against the Caco-2 cancer cell line using the MTT assay showed that compound **277** exhibited weak to moderate activity, with a half-maximal inhibitory concentration (IC_50_) of 51.6 μM [76].

Siwen Niu et al. isolated a new sesquiterpenoid, malfilanol C (**278**), from the deep-sea-derived fungus *Aspergillus puniceus* A2. Compound **278** exhibited weak antibacterial activity against *Staphylococcus aureus* ATCC 29,213 [77]. Zhong-Hui Huang et al. isolated six previously undescribed compounds, punicesterones B–G (**279**–**284**), from the deep-sea-derived fungal strain *Aspergillus puniceus* SCSIO z021. Punicesterones B and C (**279**–**280**) showed cytotoxicity and could reduce intracellular lipid accumulation. Additionally, antibacterial activity assays indicated that compounds **279**–**280** exhibited moderate antibacterial activity against five bacterial strains [45].

**Figure 18:**
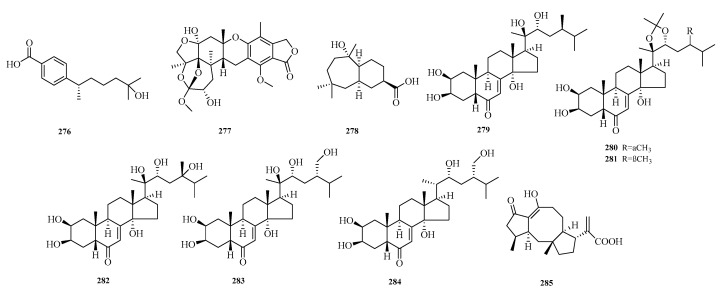
Structural diagrams of compounds **276**–**285**.

Xiuli Xu et al. isolated a new compound, aspergillusneoic acid (**285**), from the marine-derived fungus *Aspergillus brunneoviolaceus* MF180246. Antibacterial activity testing against *Staphylococcus aureus* showed that compound **285** exhibited antibacterial activity, with a minimum inhibitory concentration (MIC) of 200.0 μM [78]. The structures of compounds **276**–**285** are shown in Figure 18. 

The sources and biological activities of compounds **239**–**285** are summarized in Table 3.

### 2.4. Lignan-like Compounds

Lignan-like compounds are a class of natural organic compounds formed by the linkage of two phenylpropanoid derivatives through the β-carbon atoms of their side chains. They feature uniquely diverse structures and are widely distributed in plants including *Schisandra chinensis* and *Forsythia suspensa* [79]. Notably, their distinctive dibenzocyclooctene core structure endows them with a spectrum of biological and pharmacological activitie.

Zheng-Biao Zou et al. isolated 13 new minor furanones, including 5 pairs of enantiomers, namely (±)-nigenolides A–E (**286**–**295**) and racemic nigenolides F–H (**296**–**298**), from the deep-sea-derived *Aspergillus niger* 3A00562. Activity assays revealed that compounds **288**, **292**–**293**, and **297** could inhibit RSL3-induced ferroptosis. Among them, compound **297** exhibited half-maximal effective concentrations (EC_50_) of 0.8 μM and 0.7 μM against ferroptosis in A375 and 786-O cells, respectively. Studies indicated that compound **297** is a potential iron chelator and free radical-scavenging antioxidant, and it exerts its ferroptosis-inhibiting effect by downregulating the expression of the TXNIP gene [80].

**Figure 19:**
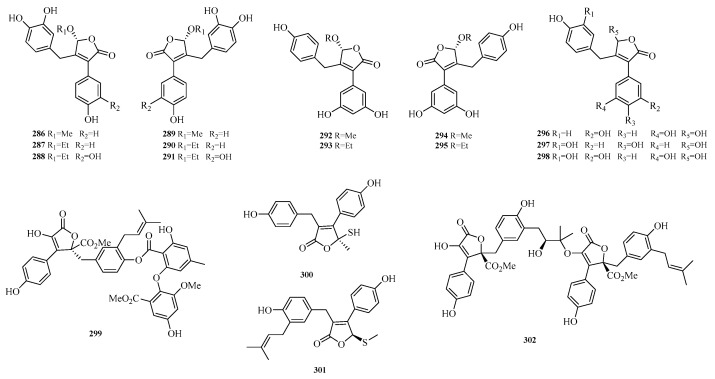
Structural diagrams of compounds **286**–**302**.

Xinwan Zhang et al. investigated the secondary metabolites of the marine-derived fungal strain *Aspergillus terreus* BTBU20211037, which was isolated from the coastal area of Qinhuangdao. A new compound, butyrolactone J (**299**), was isolated and identified therefrom. Activity testing against *Staphylococcus aureus* ATCC 25,923 showed that compound **299** exerted an inhibitory effect on it, with a minimum inhibitory concentration of 12.5 μM [81]. Guang-Yu Zhang et al. identified asperteretals L and M (**300**–**301**) from the coral-derived fungus *Aspergillus terreus*. Asperteretal M (**301**) exhibited cytotoxic activity against HCT-116 cells with an IC_50_ value of 30 μM [52]. Yufeng Jiang et al. isolated asperbutenolide A (**302**) from the marine fungus *Aspergillus terreus*. The results of its bioactivity assay showed that asperbutenolide A (**302**) had a MIC of 4.0–8.0 μM against methicillin-resistant *Staphylococcus aureus* (MRSA), indicating its potential as a novel antibacterial agent [82]. The structures of compounds **286**–**302** are shown in Figure 19. 

Hao Fan et al. isolated aspergteroids G–H (**303**–**304**) and aspergteroids I–J (**305**–**306**) from the fermented extract of the soft coral-associated fungus *Aspergillus terreus* EGF7-0-1. In myocardial protection assays, compounds **303** and **304** exhibited protective effects against tert-butyl hydroperoxide (TBHP)-induced apoptosis in H9c2 (rat cardiomyocyte) cells at low concentrations. Based on protein–protein interaction (PPI) network and Western blotting analyses, compound **303** may inhibit apoptosis and inflammatory responses in cardiomyocytes induced by TBHP, while enhancing the antioxidant capacity of cardiomyocytes [83].

**Figure 20:**
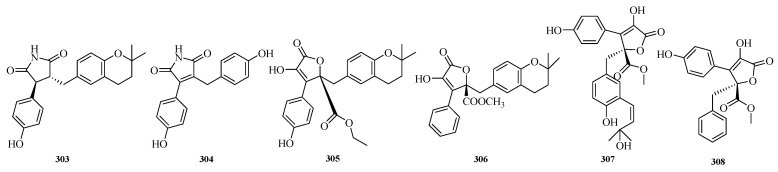
Structural diagrams of compounds **303**–**308**.

Yuwei Zhou et al. successfully discovered two new butyrolactone derivatives (**307**–**308**) from *Aspergillus terreus* GZU-31-1. The researchers tested all the isolated compounds for their anti-inflammatory effects on lipopolysaccharide-induced nitric oxide production in microglial cells (RAW 264.7 cells). The results showed that compound **307** exhibited potent anti-inflammatory activity with a half-maximal inhibitory concentration (IC_50_) of 16.3 μM, which was superior to that of the positive control indomethacin (IC_50_ = 24.0 μM) [84]. The structures of compounds **303**–**308** are shown in Figure 20. 

The sources and biological activities of compounds **268**–**308** are summarized in Table 4.

### 2.5. Cyclopeptides

Cyclopeptides are cyclic molecules formed by amino acids linked end-to-end through peptide bonds and are characterized by structural stability and diverse bioactivities. Widely distributed across animals, plants, and microorganisms, these compounds owe their unique properties to their cyclic framework: notably, this structure endows them with resistance to protease degradation and confers upon them a range of pharmacological activities.

Maokun Zheng et al. isolated a new cyclopentapeptide, cotteslosin D (**309**), from the culture of the sponge-derived fungus *Aspergillus versicolor* 2-18. Antibacterial activity testing of compound **309** showed that it exhibited weak antibacterial activity against *Escherichia coli* and *Staphylococcus aureus* [85].

Yu Wang et al. isolated four new cyclic pentapeptides, avellanins D–G (**310**–**313**), from the mangrove-derived fungus *Aspergillus fumigatus* GXIMD 03099. Activity screening results showed that compound **311** exhibited insecticidal activity against newly hatched Culex quinquefasciatus larvae, with a half-lethal concentration (LC_50_) of 86.6 μM; compound **313** showed weak activity against *Vibrio harveyi*, with a minimum inhibitory concentration (MIC) of 5.9 μM [86].

Qin Li et al. identified four new cyclic tetrapeptides, violaceotides B–E (**314**–**317**), from the sponge-associated fungus *Aspergillus insulicola* IMB18-072. Activity assay results showed that compounds **315** and **316** exhibited selective antibacterial activity against aquatic pathogens *Edwardsiella tarda* and *E. ictaluri* (*Edwardsiella ictaluri*). Furthermore, at a concentration of 10.0 μM, compounds **314**–**317** inhibited the expression of the inflammatory mediator interleukin-6 (IL-6) in lipopolysaccharide (LPS)-induced RAW264.7 cells [87].

Lu-Ping Chi et al. isolated the pentapeptides aspertides A–E (**318**–**322**) from the marine fungi *Aspergillus tamarii* MA-21 and *Aspergillus insuetus* SD-512. In bioactivity assays, compounds **321** and **322** exhibited antibacterial activity against various aquatic pathogens, including *Edwardsiella tarda*, *Vibrio alginolyticus*, *Vibrio anguillarum*, *Vibrio vulnificus*, and *Staphylococcus aureus*, with minimum inhibitory concentrations (MICs) ranging from 8.0 to 32.0 μM [88].

**Figure 21:**
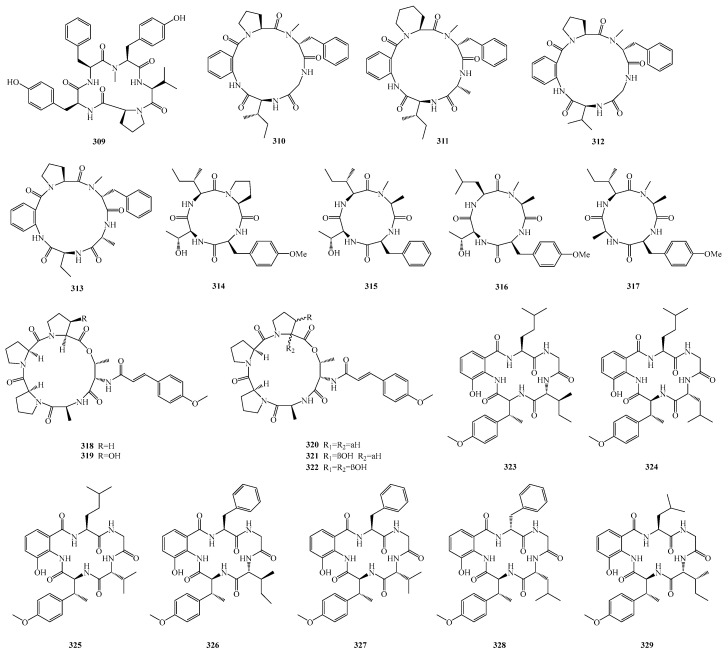
Structural diagrams of compounds **309**–**329**.

Wenjuan Ding et al. discovered seven new cyclopentapeptides, namely pseudoviridinutans A–F (**323**–**329**), from the marine-derived fungus *Aspergillus pseudoviridinutans* TW58-5. Bioassays revealed that compounds **323**–**329** possess anti-inflammatory potential; in particular, compound **328** can inhibit the production of nitric oxide (NO), a key inflammatory mediator, in lipopolysaccharide (LPS)-induced RAW264.7 murine macrophage cells by regulating the expression levels of NLRP3 and inducible nitric oxide synthase (iNOS) [89]. The structures of compounds **309**–**329** are shown in Figure 21. 

The sources and biological activities of compounds **309**–**329** are summarized in Table 5.

### 2.6. Other Types of Compounds

Sulfur-containing compounds and chlorine-containing compounds are two important classes of bioactive molecules. Sulfur-containing compounds exert antibacterial, antitumor, and other activities through their reactive sulfur atoms; chlorine-containing compounds, due to their unique polarity, exhibit prominent effects in antimalarial, antifungal, and related applications. These compounds have significant applications in fields such as biology, medicine, chemical industry, and materials science.

Muhammad Hasan Bashari et al. successfully isolated and identified two new compounds, Unguisol A and Unguisol B (**330**–**331**), from the endophytic fungal strain *Aspergillus unguis* derived from a marine sponge. These two new compounds can induce apoptosis in MDA-MB-231 breast cancer cells by downregulating BCL2L1 mRNA and cause cell cycle arrest at the S phase by downregulating AKT1 mRNA [90]. Lai-Hui Dai et al. isolated fumianthrogliotoxin (**332**) from the culture of the seawater-derived fungus *Aspergillus fumigatus* AF1 [9].

**Figure 22:**
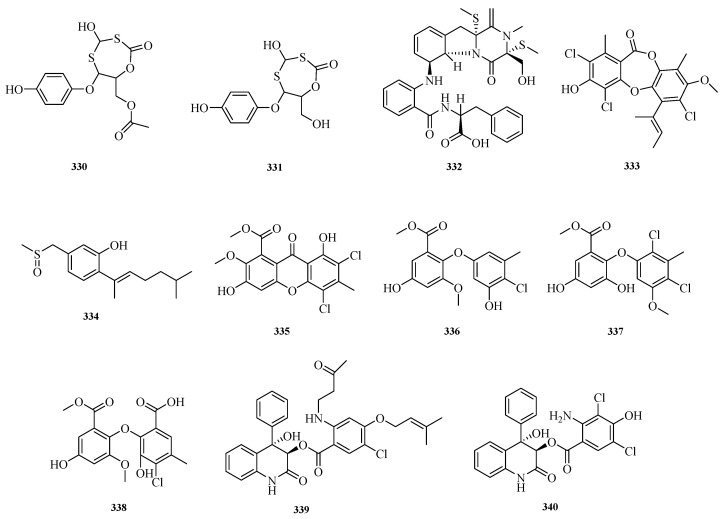
Structural diagrams of compounds **330**–**340**.

Nguyen Thi Hoang Anh et al. isolated an undescribed depsidone (**333**) from the marine sponge-derived fungus *Aspergillus nidulans* M256. Compound **333** exhibited selective biological activity against Gram-positive bacteria (MICs: 2.0–4.0 μM) and yeast (MICs: 8.0 μM) [91]. Xiao Yang et al. isolated a new sulfoxide-containing dibornyl sesquiterpenoid, aspersydosulfoxide A (**334**), from the marine-derived fungus *Aspergillus sydowii* LW09 [92]. Jia-Xin Li et al. isolated four new chlorinated biphenyls, aspergetherins A–D (**335**–**338**), from the rice fermentation product of the marine sponge-associated fungus *Aspergillus terreus* 164018. Antibacterial activity evaluation showed that compounds **335** and **337** exhibited anti-MRSA activity with minimum inhibitory concentrations (MICs) ranging from 1.0 to 128.0 μM [93]. Hu Zhibo et al. isolated and identified asperelines E and F (**339**–**340**) from the seagrass-derived fungus *Aspergillus alabamensis*. As a new natural fungicide, compound **340** exhibited moderate to potent inhibitory activity against all tested strains, including one Gram-negative bacterium *Edwardsiella ictalurid* (MIC, 10.9 μM) and four Gram-positive bacteria: *Streptococcus iniae* (MIC, 43.6 μM), *Staphylococcus aureus* (MIC, 21.8 μM), *Streptococcus parauberis* (MIC, 87.3 μM), and *Bacillus subtilis* (MIC, 21.8 μM) [42]. The structures of compounds **330**–**340** are shown in Figure 22. 

The sources and biological activities of compounds **330**–**340** are summarized in Table 6.

## 3. Results and Discussion

**Figure 23:**
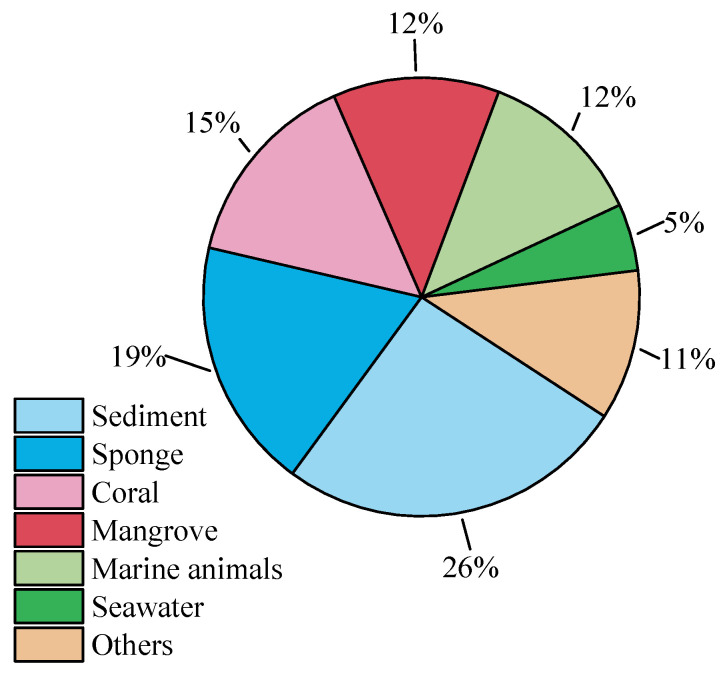
Source categories of environmental samples of marine *Aspergillus*.

Natural products represent a vital repository of bioactive substances, encompassing a diverse array of active components with distinct functions. In the research on marine *Aspergillus* species, our focus has centered on novel compounds derived from *Aspergillus* strains. This study counted 340 natural products produced by 81 *Aspergillus* strains. Analysis of the environmental sources of these strains (see Figure 23) revealed that marine sediments constitute the primary source of marine *Aspergillus*; among the total 81 strains, 21 were isolated from marine sediments. Additionally, sponges, corals, mangroves, and marine animals are also important sources of marine *Aspergillus*, with a relatively balanced distribution in the number of strains obtained from these sources. In summary, *Aspergillus* exhibits a widespread distribution in marine environments.

**Figure 24:**
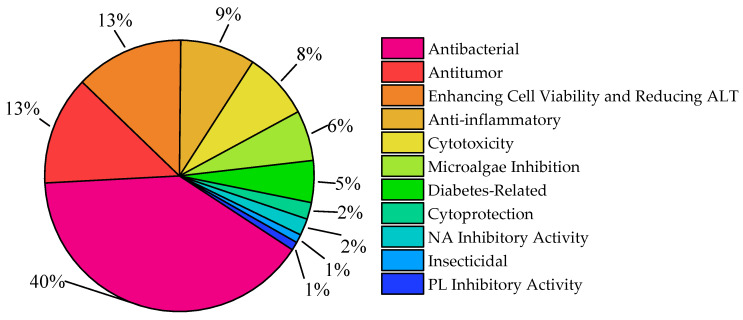
Statistical chart of compound bioactivities.

Of the 340 compounds, only 100 exhibit bioactivity, and Figure 24 is a statistical chart showing the classification of the bioactivities of these 100 compounds. Antibacterial activity is dominant at 40%, followed by antitumor activity and “enhancing cell viability & reducing ALT in mouse hepatocytes” (each 13%). Medium-proportion types include anti-inflammatory (9%), cytotoxicity (8%), microalgae inhibition (6%), and diabetes-related (5%) activities. Low-proportion ones are cytoprotection/NA inhibitory activity (each 2%) and insecticidal/PL inhibitory activity (each 1%), reflecting a focus on antibacterial functions alongside diverse research in disease treatment, cell protection, and ecological regulation.

Based on the statistical results mentioned above, among the various compounds produced by marine *Aspergillus* in recent years, alkaloids and polyketides have shown the most prominent yields, accounting for nearly two-thirds of the total number of compounds. It can thus be inferred that *Aspergillus* is highly likely to have advantages in producing alkaloids and polyketides, although this inference requires further verification. In subsequent studies on specific strains, it was found that most of the compounds they produce belong to polyketides, which initially aligns with the previous inference.

Inflammation is essential for the body’s defense yet causes tissue damage when uncontrolled and it acts as a key manifestation of diseases such as arthritis, asthma, and cardiovascular conditions [94]. While existing anti-inflammatory drugs (e.g., NSAIDs, glucocorticoids) alleviate symptoms, they have limitations like gastrointestinal or renal side effects—driving the search for safer alternatives [95], with fungal-derived natural products emerging as a promising resource [96]. However, among 81 analyzed studies, only 8 assessed the anti-inflammatory properties of 50 new *Aspergillus*-derived compounds, and merely 9 showed potential for development into inflammation-targeted drugs. These 9 promising compounds, along with their structural types, sources, and structural characteristics, are summarized in Table 7. This underscores that the exploration of anti-inflammatory active small molecules (including those from fungal and other sources like deep-sea sediments or sponges) remains insufficient; further in-depth efforts are needed to identify such active small molecules. These molecules will provide more lead compounds for anti-inflammatory drug development, ultimately advancing the progress of the anti-inflammatory drug field.

## Figures and Tables

**Table 1:** The sources and biological activities of compounds **1**–**130**.

Compound	Producing Strain	Bioactivity	Ref.
**1**–**16**	mangroves	**1**–**7**, **9**, **10** and **12**–**15** enhancing Cell Viability and Reducing ALT	[12]
**17**–**20**	seawater samples	/	[9]
**21**–**39**	marine sediments	**34** anti-inflammatory, **22**, **23**, **36**, **37** cytotoxicity	[13]
**40**–**41**	mangroves	/	[14]
**42**–**43**	sea hare-derived	/	[15]
**44**–**46**	marine sediments	/	[16]
**47**	seawater samples	**47** antitumor	[17]
**48**–**52**	seagrass	**48**–**49** antibacterial	[18]
**53**–**54**	marine mud	**53**–**54** antibacterial	[19]
**55**–**56**	salt pan	**55** antibacterial	[20]
**57**	sponge	/	[21]
**58**	sponge	/	[22]
**59**–**61**	seawater samples	**59** antitumor	[23]
**62**–**64**	marine sediments	/	[24]
**65**	sea sheath	/	[25]
**66**	marine sediments	**66** antitumor	[26]
**67**–**69**	marine sediments	/	[27]
**70**	marine sediments	/	[28]
**71**	seaweed	**71** antitumor	[29]
**72**	spongia	/	[30]
**73**–**74**	spongia	**73**–**74** antitumor	[31]
**75**–**77**	whale	**/**	[32]
**78**–**85**	coral	**78**, **79** and **84** diabetes-related	[33]
**86**–**89**	marine sediments	**86**–**87** antibacterial	[34]
**90**–**93**	coral	**92** anti-inflammatory	[35]
**94**	marine sediments	**94** antibacterial	[36]
**95**–**100**	coral	**99** antibacterial	[37]
**101**–**102**	coral	**102** antibacterial	[38]
**103**–**105**	spongia	**104** anti-inflammatory	[39]
**106**–**107**	coral	/	[40]
**108**–**112**	marine sediments	**108** and **110** antibacterial	[41]
**113**–**118**	seagrass	**115**–**116** antibacterial	[42]
**119**–**120**	marine sediments	/	[43]
**121**–**122**	ligia exotica	/	[44]
**123**	marine sediments	/	[45]
**124**–**130**	marine sediments	**126** anti-inflammatory	[46]

**Table 2:** The sources and biological activities of compounds **131**–**238**.

Compound	Producing Strain	Bioactivity	Ref.
**131**	mangroves	/	[12]
**132**	mangroves	/	[48]
**133**	coral	/	[49]
**134**–**142**	sea hare	/	[15]
**143**–**146**	coral	/	[50]
**147**–**150**	coral	/	[51]
**151**	marine sediments	/	[16]
**152**–**153**	seagrass	/	[18]
**154**–**159**	sea mud	**158**–**159** antibacterial	[19]
**160**–**161**	coral	/	[52]
**162**–**169**	seahorse	/	[53]
**170**–**175**	spongia	**174**–**175** anti-inflammatory	[54]
**176**–**177**	marine sediments	/	[24]
**178**	seawater samples		[27]
**179**–**182**	spongia	**180** diabetes-related	[55]
**183**–**185**	mangroves	**185** antibacterial	[7]
**186**–**190**	starfish	/	[56]
**191**–**193**	coral	**191** NA inhibitory activity, **192** PL inhibitory activity	[57]
**194**	spongia	/	[31]
**195**	whale	/	[32]
**196**–**198**	Johnius belangerii	/	[58]
**199**	seagrass	**199** antibacterial	[59]
**200**–**202**	marine sediments	**200** diabetes-related	[60]
**203**–**207**	marine sediments	**205** cytotoxicity	[61]
**208**–**214**	marine sediments	**214** antitumor	[62]
**215**–**235**	spongia	**215** NA inhibitory activity	[63]
**236**	sea sheath	**236** antibacterial	[64]
**237**	mangroves	/	[65]
**238**	seaweed	/	[66]

**Table 3:** The sources and biological activities of compounds **239**–**285**.

Compound	Producing Strain	Bioactivity	Ref.
**239**–**240**	coral	/	[49]
**241**	mangroves	**241** antibacterial	[68]
**242**–**251**	seawater samples	**242** and **250** antitumor	[69]
**252**–**253**	marine sediments	/	[70]
**254**–**255**	spongia	**254** anti-inflammatory	[71]
**256**–**258**	marine sediments	/	[24]
**259**	marine sediments	**259** antibacterial	[27]
**260**–**265**	onchidium struma	/	[72]
**266**	coral	/	[57]
**267**–**268**	whale	/	[32]
**269**	spongia	**269** antibacterial	[73]
**270**–**275**	seaweed	**270**–**275** microalgae inhibition	[74]
**276**	marine sediments	/	[75]
**277**	coral	**277** antitumor	[76]
**278**	marine sediments	**278** antibacterial	[77]
**279**–**284**	marine sediments	**279**–**280** cytotoxicity and antibacterial	[45]
**285**	mangroves	**286** antibacterial	[78]

**Table 4:** The sources and biological activities of compounds **286**–**308**.

Compound	Producing Strain	Bioactivity	Ref.
**286**–**298**	marine sediments	**297** antitumor	[80]
**299**	sea mud	**299** antibacterial	[81]
**300**–**301**	coral	**301** cytotoxicity	[52]
**302**	mangroves	**302** antibacterial	[82]
**303**–**306**	coral	**303**–**304** cytoprotection	[83]
**307**–**308**	onchidium struma	**307** anti-inflammatory	[84]

**Table 5:** The sources and biological activities of compounds **309**-**329**.

Compound	Producing Strain	Bioactivity	Ref.
**309**	spongia	**309** antibacterial	[85]
**310**–**313**	mangroves	**311** insecticidal, **313** antibacterial	[86]
**314**–**317**	spongia	**315**–**316** antibacterial	[87]
**318**–**322**	mangroves	**321**–**322** antibacterial	[88]
**323**–**329**	marine sediments	**328** anti-inflammatory	[89]

**Table 6:** The sources and biological activities of compounds **330**–**340**.

Compound	Producing Strain	Bioactivity	Ref.
**330**–**331**	spongia	**330**–**331** antitumor	[90]
**332**	seawater samples	/	[9]
**333**	spongia	**333** antibacterial	[91]
**334**	marine sediments	/	[92]
**335**–**338**	spongia	**335** and **337** antibacterial	[93]
**339**–**340**	seagrass	**340** antibacterial	[42]

**Table 7:** The sources and structures of compounds with inflammation-related activity.

Compound	Structural	Producing Strain	Structural Characteristics	Ref.
**34**	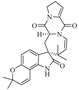	deep-sea sediment	Alkaloid	[12]
**92**	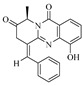	coral samples	Alkaloid	[35]
**104**	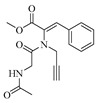	sponge	Alkaloid	[39]
**126**	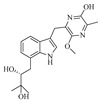	deep-sea sediment	Alkaloid	[46]
**174**–**175**	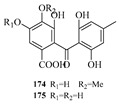	sponge	Polyketide	[54]
**254**	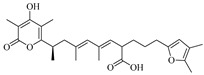	sponge	Terpenoid	[71]
**307**	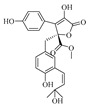	Onchidium stroma	Lignan-like compound	[84]
**328**	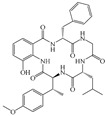	hydrothermal vent sediment	Cyclopeptide	[89]

## Data Availability

No new data were created in this study.
